# Horner Syndrome Due to Spontaneous Internal Carotid Artery Dissection

**DOI:** 10.7759/cureus.3382

**Published:** 2018-09-28

**Authors:** Nidhi Shankar Kikkeri, Elanagan Nagarajan, Ragha Chaitanya Sakuru, Pradeep C Bollu

**Affiliations:** 1 Neurology, University of Missouri, Columbia, USA

**Keywords:** miosis, ptosis, anhidrosis, carotid artery dissection, oculosympathetic pathway

## Abstract

Horner syndrome is a constellation of neurological findings consisting of ipsilateral ptosis, miosis, and anhidrosis. Partial Horner syndrome, comprising ipsilateral ptosis and miosis in the absence of anhidrosis, is a well-documented but uncommon manifestation of internal carotid artery dissection. We report the case of a 42-year-old male patient who presented with ipsilateral ptosis and miosis and was subsequently diagnosed with internal carotid artery dissection. In this case report, we discuss the anatomy of the oculosympathetic pathway and the pharmacological diagnosis for a better understanding of the localization of the lesions causing Horner syndrome.

## Introduction

Horner syndrome (HS) was first described by Francois Pourfour du Petit in 1727 but was named after a Swiss ophthalmologist Johann Friedrich Horner in 1869. HS is noted in about 25% of patients with carotid artery dissection (CAD) [1]. CAD, a well-known cause of ischemic stroke [2], commonly manifests with ipsilateral pain affecting the head, neck, or face in about 80% of patients [1]. HS is a relatively uncommon manifestation of CAD and is due to an interruption in the sympathetic chain along its course [1,3].

## Case presentation

**Figure 1:**
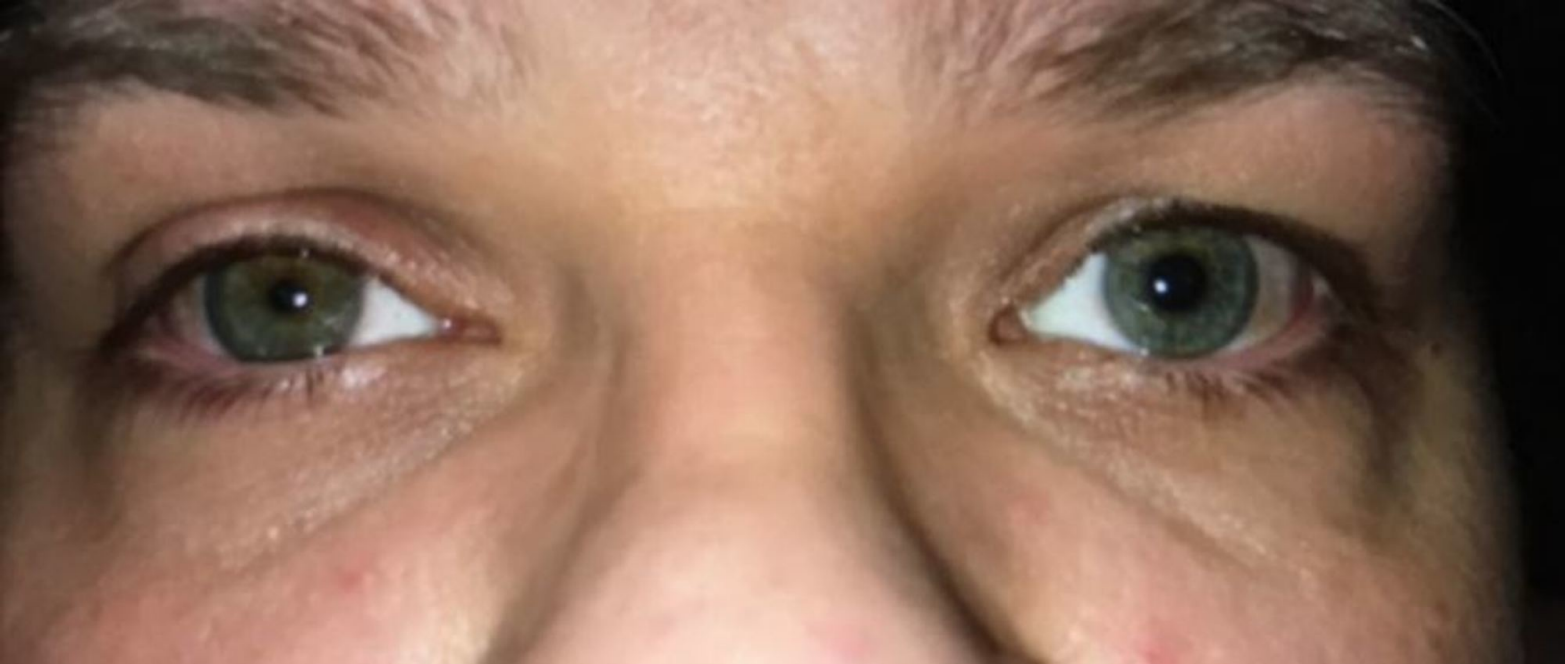
Eye examination showing miosis, partial ptosis, and enophthalmos of the right eye

**Figure 2:**
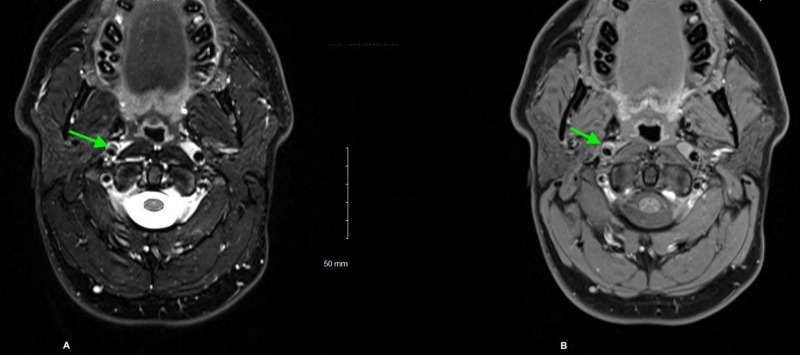
T1 axial sections of MRI neck showing a double lumen abnormality at the petrous segment of the right internal carotid artery (A) and heterogenous signal with contrast enhancement (B) suggesting right internal carotid artery dissection MRI: Magnetic resonance imaging.

**Figure 3:**
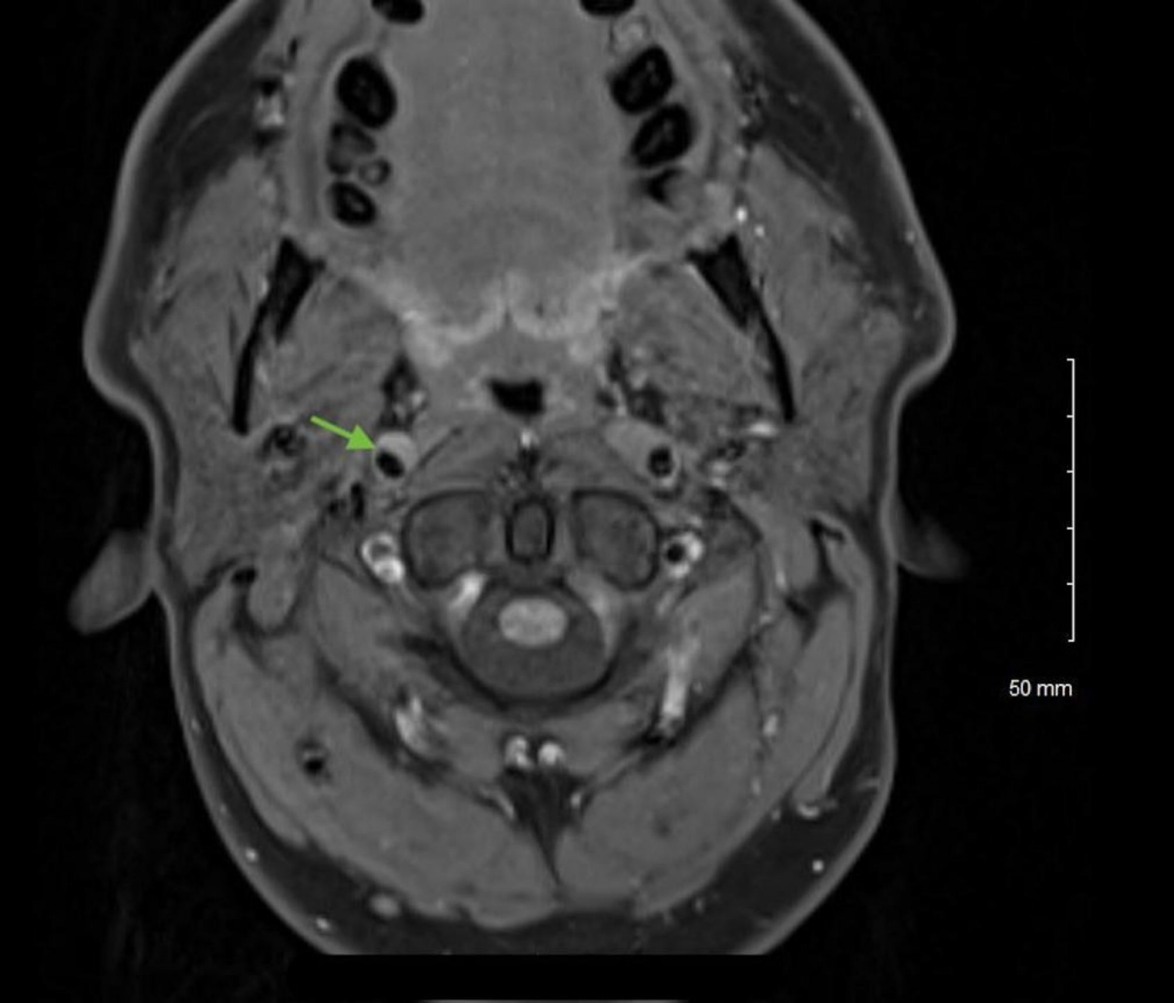
T1 axial section of MRA neck showing a double lumen abnormality at the petrous segment of the right internal carotid artery, suggesting dissection MRA: Magnetic resonance angiography

A 42-year-old male patient with a past medical history of a migraine headache presented to the emergency department after a preliminary evaluation by his primary care provider of drooping of the right upper eyelid and asymmetric pupils. The patient complained of a right-sided headache, retro-orbital pain in the right eye, and dull, aching right-sided facial pain for one day. The patient denied visual blurriness, obscurations, photophobia, or neck pain. There were no reports of recent trauma or chiropractic manipulation of his neck. On physical examination, visual acuity was 20/20 bilaterally. The ophthalmological examination was remarkable for partial ptosis and enophthalmos of the right eye (Figure 1). The pupillary examination showed anisocoria with miosis in the right eye (Figure 1) with the asymmetry more prominent in the dark. Extraocular movements were intact bilaterally. The rest of the ophthalmological and neurological examination was unremarkable. The patient was admitted to the hospital for further evaluation. Magnetic resonance imaging (MRI) of the neck (Figure 2) and magnetic resonance angiography (MRA) of the head and neck (Figure 3) revealed a dissection of the cervical and petrous segments of the right internal carotid artery. The patient was diagnosed with Horner syndrome secondary to right internal carotid artery dissection. He was then started on dual antiplatelet therapy with aspirin and clopidogrel for three months. The patient was eventually discharged after clinical improvement and asked to follow up with neurology on an outpatient basis.

## Discussion

Horner syndrome classically presents with ipsilateral ptosis, miosis, and anhidrosis [3]. Enophthalmos and loss of ciliospinal reflex may also be present [4]. HS is classified based on the anatomic location of the lesion in the oculosympathetic pathway into first-order (central), second-order (pre-ganglionic), and third-order (post-ganglionic) types [5]. Although HS is an uncommon presentation of ICA dissection, it is important to recognize it at an early stage, as the proper initiation of the treatment would potentially avoid devastating neurological sequelae like embolic strokes, aneurysmal rupture, and death [6].

Anatomy of the oculosympathetic pathway

Understanding the neuroanatomical concepts of the oculosympathetic pathway is essential for understanding the neurological findings and in localizing lesions in HS as discussed in Table 1 [4-5].

**Table 1:** Types of Horner syndrome with location and common causes

TYPES	LOCATION	CAUSES	FEATURES
First Order (Central)	Hypothalamic, brainstem, or spinal cord (C1 to T2) lesion	Cerebrovascular accidents, spinal cord injury above T2-T3, intracranial tumors, multiple sclerosis	Ipsilateral ptosis, miosis, and anhidrosis
Second Order (Preganglionic)	Lesion involving the apex of the lung, the mediastinum, or the anterior aspect of the neck	Pancoast tumor, mediastinal lymphadenopathy, trauma to brachial plexus, cervical rib injury	Ipsilateral ptosis, miosis, and anhidrosis
Third Order (Postganglionic)	Lesion involving the internal carotid artery, skull base, cavernous sinus, superior orbital fissure, and orbital apex	ICA dissection or aneurysm, carotid cavernous fistula, cluster headaches or migraines, temporal arteritis	Ipsilateral ptosis, miosis, and limited or absent anhidrosis

The oculosympathetic pathway comprises neurons of three orders. The first-order neurons extend from the hypothalamus and descend along the brainstem into the spinal cord to the first synapse at the cervical (C8) and thoracic (T1-T2) levels [7]. The second-order neurons then exit through the ventral spinal nerve roots, arch over the apex of the ipsilateral lung, and join the cervical sympathetic chain to reach the superior cervical ganglion, located near the bifurcation of the common carotid artery. The third-order neurons ascend within the adventitia of the ICA, pass through the cavernous sinus, and then join the ophthalmic division of the trigeminal nerve [8]. The oculosympathetic fibers terminate by innervating the iris dilator muscle as well as the muller muscle, which is responsible for a minor portion of upper lid elevation. The third-order neurons responsible for facial sweating are distributed via the ICA to the supraorbital artery (medial brow) and via the external carotid artery (ECA) to supply the lateral brow and the ipsilateral face [9]. Hence, the presence of anhidrosis along with ptosis and miosis localizes the lesion to first-order or second-order neurons (pre-ganglionic). When ipsilateral ptosis and miosis are seen without anhidrosis (partial HS), as in our patient, a post-ganglionic cause should be suspected. A lack of anhidrosis reflects the sparing of sudomotor fibers that travel adjacent to the ipsilateral ECA [5].

Pharmacological diagnosis and localization of Horner syndrome

Several agents have been used in confirming the diagnosis and in localizing the anatomical lesion in patients with Horner syndrome. Common drugs used are cocaine, hydroxyamphetamine, and apraclonidine.

Topical Cocaine Test

Two percent to 10% topical cocaine is used in confirming the diagnosis of HS. Cocaine is a mydriatic and acts by inhibiting the reuptake of nor-epinephrine at postganglionic sympathetic nerve endings. The failure of pupillary dilatation indicates sympathetic denervation thereby confirming the diagnosis of HS [10].

Topical Hydroxyamphetamine Test

The topical application of 1% solution causes pupillary dilation by releasing endogenous norepinephrine from post-ganglionic sympathetic nerve endings. A failure of pupillary dilation indicates a post-ganglionic lesion and, hence, it is used in localizing the lesion to the third-order neurons [7].

*Topical Apraclonidine Test*​​

Apraclonidine is an alpha agonist and can be used in confirming the diagnosis of HS. A topical application of 1% apraclonidine causes a dilatation of the pupil by acting on alpha receptors, which are upregulated in patients with sympathetic denervation, whereas the unaffected pupil is fairly insensitive or dilates less than 0.5 mm to the topical application of apraclonidine. This causes a reversal of anisocoria with the miotic pupil in patients with HS, dilating more than the size of the normal pupil [10]. Apraclonidine is slightly less sensitive than cocaine and false negative results are usually seen five to eight days after the onset of HS until the up-regulation of the alpha receptors.

Our patient presented with a unilateral headache, facial pain, and a drooping of the right upper eyelid. The physical examination findings of ipsilateral ptosis and miosis, in the absence of anhidrosis, helped us in localizing the etiology of HS to third-order neurons.

Though HS is a well-documented manifestation of carotid dissection, the association is often overlooked [11]. In the past, HS was rarely attributed to ICA dissection. One of the studies, which had reviewed 450 cases of HS, reported that tumor (13%) and cluster headache (12%) were the most frequent causes of the syndrome [12]. With the advent of MRI, carotid dissection is being recognized more frequently as a cause of HS [11]. A prospective study by Digre et al., in which MRI was performed in 33 patients with established HS, found that 15% of patients had a carotid artery dissection confirmed with angiography [13].

The early diagnosis and prompt treatment of carotid artery dissection are essential to prevent cerebral infarction and the progression of neurological symptoms [11]. Treatment options for ICA dissection include anticoagulation, antiplatelet therapy, surgery, and observation [11].

## Conclusions

ICA dissection should be suspected in a patient with partial HS. Occasionally, HS can be the only manifestation in ICA dissection. A high index of suspicion and a careful and detailed ophthalmologic examination is necessary to uncover this relatively uncommon manifestation of ICA dissection. Knowledge of the basic anatomy involved in HS helps in localizing the lesion. Early diagnosis helps in the prompt initiation of treatment, which can prevent the devastating complications of carotid artery dissection.
